# Pathology of Urologic Cancers

**DOI:** 10.3390/cancers14153751

**Published:** 2022-08-01

**Authors:** Giovanni Tossetta, Roberta Mazzucchelli

**Affiliations:** 1 Department of Experimental and Clinical Medicine, Università Politecnica delle Marche, 60126 Ancona, Italy; 2 Department of Biomedical Sciences and Public Health, Section of Pathological Anatomy, Università Politecnica delle Marche, 60126 Ancona, Italy

We are pleased to present this Special Issue of *Cancers*, entitled “Pathology of Urologic Cancers”. Urologic cancers include kidney, bladder and prostate cancer, and are in the top-ten list of human cancers. The incidence of urologic malignancies has increased significantly over the last 50 years [1]. Despite advances in medical tumor therapy, the occurrence of chemoresistance and metastatic disease is a common cause of death in patients with urological malignancies [2,3,4]. The pathogenetic mechanisms of kidney, bladder and prostate tumors are very different and represent an important challenge for clinical teams, requiring close collaboration among urologists, oncologists and pathologists [5,6,7,8]. Moreover, the expression level of specific proteins that are detectable by immunohistochemistry and the use of molecular biomarkers are reported to be helpful in predicting the recurrence, progression and development of metastasis. For these reasons, novel biomarkers are always needed in clinical practice for patient risk stratification and personalized therapy to develop new therapeutic approaches that can improve diagnosis and treatment outcomes [9,10]. To this end, we require a better understanding of the molecular changes that occur in urological tumors and the development of molecular biomarkers that are able to predict tumor behavior and the risk of disease recurrence and chemoresistance.

In recent decades, intense research efforts have focused on identifying novel biomarkers (DNA, microRNAs, lncRNAs, proteins, etc.), which could be used for diagnosis [11,12,13,14], prognosis [15,16,17,18], or as a therapeutical target [19,20,21,22], with an important impact on neoplastic diseases [23,24].

Thus, finding useful biomarkers that can allow for the early and appropriate treatment of urologic malignancies represents a key point to improve the outcome of these diseases.

The usefulness of specific biomarkers could help to increase understanding of the complexity of some urologic tumors with very heterogeneous malignancy [25,26]. In renal cancer, particularly clear cell renal cell carcinomas (CCRCC), VHL mutation or methylation and chromosomal gains and losses are considered tumor initiators and have lethal potential for the neoplasms [27]. Moreover, it has been reported that exosomes, small membrane vesicles secreted by cells into the extracellular space, are involved in tumorigenesis and can be used to distinguish muscle-invasive bladder cancer from its non-invasive counterparts [28]. Furthermore, patients harboring germline mutations in specific genes (e.g., BRCA1/2) showed an increased chance of developing aggressive prostate cancer with a high mortality rate [29].

In addition, the metabolism of neoplastic cells and the microenvironment play an important role in the development and progression of cancer.

Cancer has been often associated with chronic inflammation and oxidative stress [30,31,32]. Oxidative stress plays a key role cancer development, including urologic cancers [33]. The production of reactive oxygen and nitrogen species (ROS/RNS) is normally associated with physiological processes such as oxidative respiration. When the rate of ROS/RNS generation is balanced by the scavenging activity of antioxidant compounds, e.g., glutathione (GSH), the redox homeostatic balance is maintained. In the case of elevated ROS levels, the latter cannot be counteracted by the cellular antioxidant response, causing a redox imbalance that results in oxidative injures to cell organelles [34], leading to the development of several diseases, including cancer [30].

Natural products (often called phytonutrients) are biological compounds present in plants (e.g., carotenoids, anthocyanins and flavonoids) as well as in bacteria, fungi and marine organisms. Many of these compounds are used as cancer adjuvants, since they can attenuate chemotherapeutic resistance and also counteract some side effects of these chemotherapeutic agents. Moreover, several natural compounds have recently been proposed as immunity regulators in various cancer types since they can regulate the activity of many immune cell types, including T and B lymphocytes, natural killer (NK), Treg and dendritic cells. In this way, these compounds can regulate cytokine production in the tumor microenvironment, regulating cancer cell growth and proliferation [35].

Understanding the mechanisms involved in the regulation of urological cancer development/progression and the main modulators involved in the activation/inhibition of the pivotal signaling pathways in these malignancies can lead to new perspectives in the treatment of these pathologies. Thus, the aim of this Special Issue is to provide an overview of the molecular and signaling alterations involved in urological cancer’s development, diagnosis and treatment.
